# Efficient Processing of Geospatial mHealth Data Using a Scalable Crowdsensing Platform †

**DOI:** 10.3390/s20123456

**Published:** 2020-06-18

**Authors:** Robin Kraft, Ferdinand Birk, Manfred Reichert, Aniruddha Deshpande, Winfried Schlee, Berthold Langguth, Harald Baumeister, Thomas Probst, Myra Spiliopoulou, Rüdiger Pryss

**Affiliations:** 1 Institute of Databases and Information Systems, Ulm University, 89081 Ulm, Germany; ferdinand@birk-home.de (F.B.); manfred.reichert@uni-ulm.de (M.R.); 2 Department of Clinical Psychology and Psychotherapy, Ulm University, 89081 Ulm, Germany; harald.baumeister@uni-ulm.de; 3 Department of Speech-Language-Hearing Sciences, Hofstra University, Hempstead, NY 11549, USA; aniruddha.deshpande@hofstra.edu; 4 Clinic and Policlinic for Psychiatry and Psychotherapy, University of Regensburg, 93053 Regensburg, Germany; winfried.schlee@gmail.com (W.S.); berthold.langguth@medbo.de (B.L.); 5 Department for Psychotherapy and Biopsychosocial Health, Danube University Krems, 3500 Krems, Austria; thomas.probst@donau-uni.ac.at; 6 Department of Technical and Business Information Systems, Otto-von-Guericke-University Magdeburg, 39106 Magdeburg, Germany; myra@ovgu.de; 7 Institute of Clinical Epidemiology and Biometry, University of Würzburg, 97080 Würzburg, Germany; ruediger.pryss@uni-wuerzburg.de

**Keywords:** mHealth, crowdsensing, tinnitus, geospatial data, cloud-native, stream processing, scalability, architectural design

## Abstract

Smart sensors and smartphones are becoming increasingly prevalent. Both can be used to gather environmental data (e.g., noise). Importantly, these devices can be connected to each other as well as to the Internet to collect large amounts of sensor data, which leads to many new opportunities. In particular, mobile crowdsensing techniques can be used to capture phenomena of common interest. Especially valuable insights can be gained if the collected data are additionally related to the time and place of the measurements. However, many technical solutions still use monolithic backends that are not capable of processing crowdsensing data in a flexible, efficient, and scalable manner. In this work, an architectural design was conceived with the goal to manage geospatial data in challenging crowdsensing healthcare scenarios. It will be shown how the proposed approach can be used to provide users with an interactive map of environmental noise, allowing tinnitus patients and other health-conscious people to avoid locations with harmful sound levels. Technically, the shown approach combines cloud-native applications with Big Data and stream processing concepts. In general, the presented architectural design shall serve as a foundation to implement practical and scalable crowdsensing platforms for various healthcare scenarios beyond the addressed use case.

## 1. Introduction

Nowadays, smartphones can be considered as an everyday object. Along this trend, many other trends have been fanned. For example, as many people become more and more health-conscious, smartphones can be utilized to support the trend to monitor someone’s own health status. Mobile crowdsensing is one technology that is often used in this context. With mobile crowdsensing, a person’s health status can be efficiently monitored, but beyond this opportunity, each crowd user benefits from the measurements of other crowd users as well since measured values can be compared among the users over time. Especially for users that suffer from a chronic disease or disorder, the approach of mobile crowdsensing can be very helpful [1,2,3]. For the tinnitus chronic disorder, the measurement of noise exposure can help each person in a crowd to avoid harmful locations based on the individual measurements and their aggregations. For example, if a defined threshold in terms of the average loudness is exceeded for a certain location, tinnitus users can be warned to avoid these places and therefore avoid an unhealthy noise exposure.

Importantly, smartphones with their built-in sensors are able to reliably measure the noise level of a user’s surrounding and store these measurements to a backend, which calculates average values and responses with the aggregated data. Based on this information, places with an unhealthy noise exposure can be, for example, visually highlighted on a map on the smartphone of a user. On the one hand, as potentially millions of noise measurements have to be collected and processed continuously and concurrently, such a backend in the mHealth context has to ensure a high degree of scalability to avoid a degradation of the service for its users [3]. In addition, since the workload of such a system can change frequently (e.g., due to day times or public events), such a backend should also provide elasticity. On the other hand, efficient computations on geospatial data are non-trivial and choosing an adequate data representation format can be a complex task. Furthermore, the architecture of such a system should allow for flexible technical changes. Ensuring flexibility can become important, for example, if a mobile operating system changes the sensor features used for measurements or other additional requirements emerge. Therefore, monolithic backends using relational databases are usually not sufficient if crowdsensing is used to store, process, and deliver stream-based smartphone data. When working on a scenario like the measurement of noise levels, which is considered in the work at hand, we propose that new architectural designs become necessary. For this purpose, we discuss a mobile crowdsensing architecture, which is based on (1) cloud-native applications as well as (2) Big Data and stream processing concepts. This work discusses the newly conceived architectural design and also shows an implemented prototype on top of the architecture, which enables the aforementioned noise level measurements. The architecture, its design principles, and the prototype show that for tinnitus patients, the compiled approach is feasible in particular. However, the proposed approach can be used for other healthcare scenarios with similar requirements as well.

As health-conscious people, especially those suffering from a chronic disease, crave technical applications like those shown here; the overarching goal of this work is to utilize the power of the crowd on one hand, but provide a scalable and flexible technical solution on the other hand. To summarize the pursued aspects, the contributions of our work are as follows:

- Practically, geospatial data that are gathered by the smartphones of crowd users shall be the basis to provide a noise exposure map for tinnitus patients and the general public. This map shall help patients and other health-conscious people to avoid unhealthy places.
- Technically, a cloud-native architectural design based on microservices and stream processing has been composed, which is able to collect, process, aggregate, store, and deliver measurement data in mobile crowdsensing scenarios in an efficient and scalable manner. In this context, it is shown how the mobile context is captured by the proposed architectural design in such a way that it is able to cope with potentially increasing amounts of workloads without a noticeable degradation of the resulting response times for its users.
- Generally, the technical setting shown in this paper shall help other researchers to create scalable mobile crowdsensing solutions for various healthcare scenarios.

Moreover, recent works show [3,4] that approaches that combine contemporary mobile technology with paradigms from the healthcare side should address the question how this combination can be accomplished so that various diseases and their particular aspects can be flexibly addressed. To this end, based on the architecture that is presented in this work, including the promising experimental results, the approach may constitute as a solid basis for other disorders than tinnitus. For example, for the management of stress, noisy places should be also avoided. In addition, stress constitutes generally a negative factor and is also being negative for many diseases and disorders, including tinnitus. Therefore, the measurements done in this work may be beneficial for other scenarios. As stress is also being important for companies and the management of employees, the solutions can also be utilized in non eHealth scenarios. Finally, the presented approach can be adapted to other measurements like, for example, the aggregated gathering of weather-related factors. The latter can then be used, for example, for the better management of migraine.

In summary, when designing a system in such a context, the following technical challenges must be addressed:

- **Scalability and Elasticity**: The system should be able to cope with increasing workloads without significant loss of performance and adapt its resources accordingly.
- **Efficient Geospatial Processing**: The system should be able to efficiently process geospatial data and store it in an adequate data representation format.
- **Flexibility**: The system should allow flexible changes in order to cope with changing requirements.

**Figure 1:**
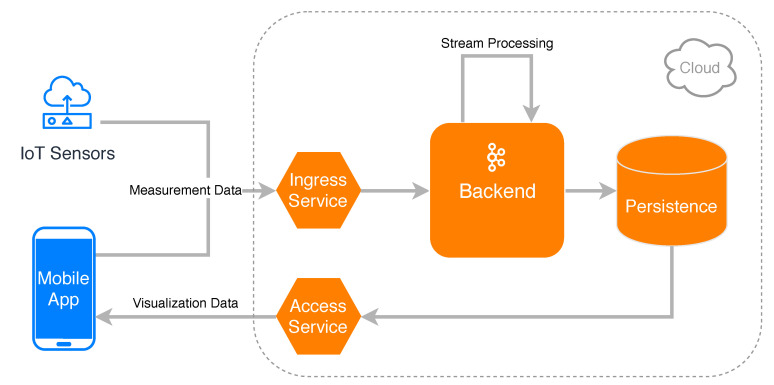
System design overview of the crowdsensing platform.

An overview of the resulting system design that addresses these challenges is shown in Figure 1. A mobile application as well as Internet of Things (IoT) sensors are able to contribute their measurement data by sending the data to an ingress service in the cloud. The measurements of all users in a certain area are then processed in the backend by utilizing stream processing techniques. More specifically, the received measurements are continuously aggregated and prepared for being visualized on the smartphone of the crowd users, before finally being stored in a database. The mobile application can then request the aggregated data by querying an access service and displaying it directly on the device. The shown architecture distinguishes itself from others (like, for example, TrackYourTinnitus [2]) as it allows for the efficient processing of data from various input sources (e.g., IoT sensors and mobile applications) in the mHealth context by making use of stream processing concepts, which are embedded in a cloud-native design.

This work is an extension of the following conference paper [5]. It substantially extends the conference paper by the following aspects. First, the used data model for the representation of the geospatial data as well as the indexing mechanisms for its storage are discussed (see Section 5). Second, the stream processing as well as other implementation aspects in the Measurement Context are described in more detail (see Section 6). Third, the implemented prototype of the architecture is presented in more detail (see Section 7). Fourth, the performance and scalability of the conceptual architecture were evaluated by conducting load tests on a running instance of the developed prototype (see Section 8). Finally, related work and background information are discussed more extensively.

The work at hand is built upon the following structure. In Section 2, related work is discussed, while Section 3 introduces relevant background information. The proposed architecture is presented in Section 4, followed by a discussion in Section 5 that shows in what way geospatial data is captured and processed by the architecture. The measurement context, which is a peculiarity of the approach, is separately discussed in Section 6. Selected insights into the prototypical implementation are presented in Section 7. The experiments conducted in the scope of the performance evaluation of the architecture and their results are presented in Section 8, before concluding the paper with a summary and an outlook in Section 9.

## 2. Related Work

Mobile crowdsensing platforms that are used for urban sensing and collaborative noise maps have been already discussed. In order to enhance the spatial and temporal data resolution of noise pollution in cities, the authors of [6] implemented a participatory sensing approach, making use of an Android application and an urban sensing platform. Importantly, the smartphones’ microphones and GPS-sensors are utilized by the mobile application to perform location-related noise measurements. Following this, the collected data is transferred to the urban sensing platform. Furthermore, users are enabled to access this information basis and generate real-time noise maps or data graphs. The authors of [7], on the other hand, realize a noise monitoring platform and acoustic urban planning in smart cities by leveraging crowdsensing based on an Android application and Open Source data collection and processing techniques. Noise reduction interventions are recommended to urban planners in order to enable them to comply with European laws and regulations, using a web-based visualization application. However, none of these platforms offers an interactive visualization of the measured noise data directly on the mobile application, which would presuppose that the respective architecture is designed for mobile clients. Moreover, these approaches do not set their main focus on efficiency and scalability.

Crowdsensing platforms and architectures that address scalability and efficiency have also been considered in the past. The authors of [8] propose the middleware infrastructure MECA (mobile edge capture and analysis middleware for social sensing applications). It is designed for mobile data collection of crowdsensing applications in an efficient, flexible, and scalable manner. The common infrastructure allows for the collection of real-time data for different kinds of applications simultaneously. By introducing a high-level abstraction of phenomena to be measured, applications can express diverse data needs. Additionally, data can be shared among different applications with common information needs. Primitive data processing can be performed on the edge of the network (e.g., base stations in cellular networks). The platform CAROMM (context-aware real-time open mobile miner), proposed by the authors of [9], supports data collection for mobile crowdsensing applications by leveraging real-time mobile stream mining. This reduces the amount of sent data as well as energy-usage on mobile devices, while providing comparable accuracy to conventional approaches on the other. Different types of stream data can be captured on mobile devices, processed, managed, analyzed, and finally queried by mobile users. The collaborative mobile sensing platform MOSDEN (mobile sensor data engine) [10] enables us to capture and share sensed data between multiple distributed (mobile) applications. Its design goals are ease of use, ease of development and deployment, scalability and performance, ease of access to both on-board and external sensors, support for on-board data analytics and collaboration, as well as data sharing. In contrast to CAROMM, MOSDEN separates data collection, processing, and storage from the domain-specific application logic by providing standardized interfaces in order to reduce complexity as well as to ease the re-usability opportunities for developers. CARDAP [11], a context-aware real-time data analytics platform, deals with energy-efficient and context-aware distributed mobile data analytics in the context of distributed applications like crowdsensing. It utilizes a standardized component-oriented approach in order to provide the application-specific analytics and additionally addresses local data storage and processing in fog and cloud environments. The authors of [12] propose an approach for mobile crowdsensing based on the cloud-based publish/subscribe middleware CUPUS (cloud-based publish/subscribe middleware). The platform is able to acquire sensor data from mobile devices in a flexible and energy-efficient manner, and to perform near real-time processing of Big Data streams. It allows us to manage mobile sensor resources within the cloud, filtering, and aggregating sensor data on mobile devices, before they are transmitted to the cloud based on global data requirements, and to send push notifications from the cloud to mobile devices. However, none of the approaches discussed focuses on the mHealth context and therefore do not consider aspects such as privacy. In addition, from a technical point of view, none of these approaches combines cloud-native and stream processing concepts to enable efficient and (horizontally) scalable processing of measurement data, as proposed in the work at hand.

Platforms that deal with efficient processing of data streams have already been discussed in the literature. For example, Microsoft StreamInsight [13] is an extensible stream processing platform that enables continuous query processing, while ensuring a well-defined temporal model over incoming events. Its extensibility infrastructure allows us to process geospatial data with the help of the SQL Server Spatial library [13]. Furthermore, other approaches for processing geospatial data in particular have been considered in the past. The authors of [14] review recent developments in the context of crowdsourcing of geospatial data in particular. They identify two basic technologies that facilitate these developments: geo-referencing (e.g., GPS) and the Web 2.0 to enable user-generated content (e.g., by uploading data via broadband communication). Challenges and opportunities of geospatial big data are discussed in [15]. The authors highlight the emerging opportunities through the advancements of sensor and communication technologies as well as mobile devices and highlight the importance of high performance computing in this context. As an example, the XML-based system G-Portal [16] has been designed to organize and manage geospatial as well as geo-referenced information in order to make it available through a web search and an interactive map. Other geospatial applications like GeogDL [17] have been built on top of G-Portal.

Crowdsensing of geospatial data has already been considered in other application domains, such as vehicular networks [18,19] or location-based games [20,21]. Furthermore, the feasibility of crowdsensing platforms in the mHealth context that support people suffering from chronic disorders, especially tinnitus, has already been shown by [1,2,3,22]. However, to the best of our knowledge, none of these approaches combines efficient and scalable processing of geospatial data with crowdsensing in the mHealth context, as it is done in this work.

## 3. Background Information

Mobile crowdsensing (MCS) is a paradigm that is increasingly utilized in the mHealth context [22,23]. It has been shown that MCS has the potential to reveal meaningful medical insights when it is combined with Ecological Momentary Assessments (EMA) [24]. For instance, the authors have developed the mobile crowdsensing platform TrackYourTinnitus (TYT), which is designed for patients with the tinnitus disease [22]. Note that the tinnitus disorder can be described as the phantom perception of a sound. The related symptoms of patients are subjective and vary over time. In order to monitor and evaluate this variability of symptoms over time, TYT was realized based on EMAs and mobile crowdsensing. Notably, with a prevalence rate of 10–15% of the population worldwide, tinnitus is a chronic disorder with a high economic burden. Since there is no general treatment, patients search for valuable experiences or methods to better manage their symptoms in daily life. One possible countermeasure constitutes the avoidance of noisy places, as it is often reported that patients suddenly get a tinnitus episode or that their already existing symptoms worsen after they visited a concert. In addition, also for other phenomena like stress management, the avoidance of noisy places is recommended. Therefore, in this work, the power of the crowd shall be leveraged to create noise level maps of a region. More precisely, users should be able to measure the current noise level of their environment with their smartphones using mobile crowdsensing techniques. Noise levels maps that are created utilizing this data can be used by tinnitus patients to avoid noisy places (if the collected data is reliable). Or, being utilized by other users for other healthcare questions like the reduction of stress.

Although this work exploits the power of the crowd, it technically differs greatly from TrackYourTinnitus with respect to three major issues: First, the platform should be able to process many concurrent requests for incoming and outgoing measurement data without a significant performance loss. Hence, it should be scalable and, in the best case, also be elastic. To get a better idea of these two criteria, the following definitions for *scalability* and *elasticity* from the literature are used.

**Definition** **1.** *Scalability is “the ability of a system to maintain the satisfaction of its quality goals to levels that are acceptable to its stakeholders when characteristics of the execution environment (“the world”) and design (“the machine”) vary over expected ranges.” [25,26]*

**Definition** **2.** *“Elasticity is the degree to which a system is able to adapt to workload changes by provisioning and deprovisioning resources in an autonomic manner, such that at each point in time the available resources match the current demand as closely as possible.” [27]*

Second, the platform should be able to efficiently process, store, and deliver a large amount of geospatial data. Geospatial data or geographic data denote data with “implicit or explicit reference to a location relative to the Earth” [28]. Finding an adequate representation format for this kind of data is a key task in the design phase of such a platform. In general, computations on geospatial data are complex as operations on high-resolution coordinates, that are needed in order to aggregate geographically and hierarchically related data, are costly [29]. Therefore, it is of utmost importance to select an efficient approach for indexing and aggregating geospatial data. Third, the platform should be designed in a generic way, so that it is able to process different types of geospatial crowdsensing data (e.g., noise pollution, air pollution, or traffic information) as well as different types of utilized sensors (e.g., smartphone or stationary sensors).

To conclude, designing a crowdsensing platform that is able to collect, process, store, and deliver data for noise measurements comes with several new challenges compared to other crowdsensing solutions of the authors in particular and other existing work in general.

## 4. Technical Approach

The core functions that were identified for the mobile crowdsensing platform, that enables the implementation of a noise level map for tinnitus patients, are shown in Table 1. After the requirements analysis and during the design phase, it was decided to decompose the system into bounded contexts. The term originates from domain-driven design (DDD) and “delimits the applicability of a particular model, so that team members have a clear and shared understanding of what has to be consistent and how it relates to other contexts” [30]. The contexts serve as the inner boundaries for a global domain (e.g., crowdsensing of geospatial data), and are the result of a strategic decomposition of large components into smaller, more coherent components [31]. After defining these contexts, respective microservices were developed that can be flexibly adapted or replaced if the requirements of a context change. To this end, the five bounded contexts User Identity, Social, Measurements, Incentives, Communication, and Sensors were identified. Following this, functions were mapped to one of these contexts as shown in Table 1. Finally, one or more microservices compose a bounded context, as different patterns must be supported to technically implement a bounded context through microservices.

A cloud-native approach was selected in order to enable an efficient and scalable processing of concurrent requests for noise measurements. A cloud-native application (CNA) denotes an application that is explicitly designed to be deployed in the cloud. To this end, such applications are distributed, horizontally scalable, and elastic by design. Moreover, CNAs are composed of microservices, with a minimum of isolated states [32]. The developed prototypical implementation uses several microservices, utilizing Docker (https://www.docker.com/) as container technology and Kubernetes (https://kubernetes.io/) as container-orchestration system. Notably, in order to enable decoupled processing of incoming geospatial data, the cloud-native approach was combined with stream processing. Stream processing denotes a programming paradigm. in which data from an unbounded (i.e., infinite and ever growing) dataset (data stream or event stream) is continuously read and processed. This ongoing processing is taking place in a continuous, asynchronous, and non-blocking manner [33]. Furthermore, due to its compatibility to Apache Kafka (https://kafka.apache.org/), the library Kafka Streams was used for the implementation of the stream processing. Apache Kafka is a distributed stream processing platform that allows users to publish and subscribe to streams of messages. In Kafka, services can act as producer and publish different messages to Kafka Topics. Every interested service can then act as a consumer that subscribes to these topics and reads the respective messages [33].

**Figure 2:**
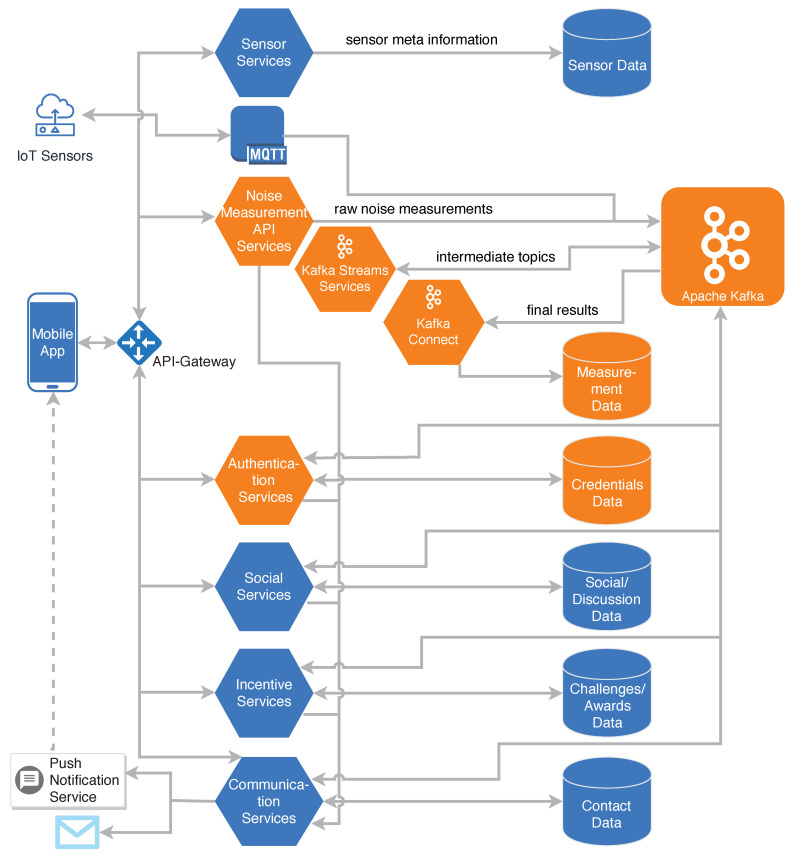
Architecture of the crowdsensing platform. The components depicted in orange are part of the proof-of-concept implementation. Reproduced with permission from Kraft et al., In Proceedings of the 2019 IEEE 32nd International Symposium on Computer-Based Medical Systems (CBMS); published by IEEE, 2019 [5].

Figure 2 shows the overall architecture of the platform. Incoming measurements from smartphones are handled by the central Measurement API Services. Measurements are then forwarded by these services to the stream processing with Apache Kafka. In addition, any type of an Internet of Things (IoT) sensor can use the message queuing telemetry transport (MQTT) protocol in order to directly contribute measurement data to Apache Kafka. Furthermore, the measurement API services provide an interface for the other services that allows them to consume raw or transformed measurement data. In Section 6, this process is described in detail. User authentication and authorization for all access-restricted services are handled by the Authentication Services. Different autonomous services manage their individual databases for sensor data, social and discussion data, incentive (i.e., challenges and awards) data, and finally communication (i.e., contact) data.

## 5. Representation of Geospatial Data

In order to efficiently transmit, store, and process geospatial data, it has to be represented by an adequate data format. A coordinate referencing system (CRS) or spatial reference system (SRS) [34] is a system that describes how entities are located in space based on their coordinates. It allows us to unambiguously identify any point in a geographical space (e.g., on Earth). In this context, an object is related to a coordinate system by a geodetic datum. One of the most known geodetic datums is the World Geodetic System (WGS). Its latest revision WGS84 [35] is used by the Global Positioning System (GPS) [36], which, in turn, is commonly used for navigation purposes. WGS approximates the sea-level of the earth by using a defined ellipsoid. A point is then described by latitude and longitude angles on this surface.

**Figure 3:**
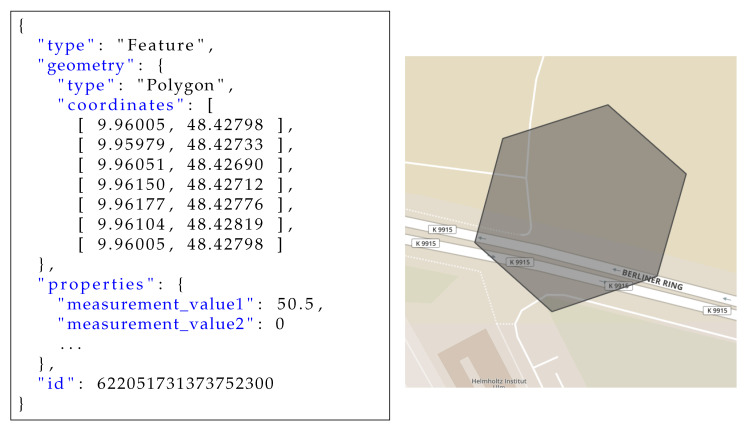
Example for GeoJSON feature object, including a polygon geometry and its visualization (Screenshot from http://geojson.io).

Furthermore, in order to provide interoperability between systems, we decided to use the OGC Simple Feature Access Specification [37], published by the Open Geospatial Consortium (OGC). It defines a common architecture on how geometric objects that are associated with a spatial reference system can be stored and accessed. Its geometry model includes definitions for geometries like points, lines, and polygons. GeoJSON [38] is a geospatial data interchange format based on the JavaScript Object Notation (JSON) that uses WGS84 as coordinate reference system and encodes geographic data structures according to the simple feature access specification. A GeoJSON object may not only represent a region of space (i.e., a Geometry), but also additional properties forming a spatially bounded entity (i.e., a Feature), or a list of features (i.e., a FeatureCollection). A feature object contains an Identifier (id), a geometry object, and additional properties that describe the entity. Geometry objects are of type Point, LineString, Polygon, MultiPoint, MultiLineString, MultiPolygon, or GeometryCollection, and are described by coordinates, which are encoded as arrays in the form [longitude, latitude], (and optionally altitude/elevation). The properties attribute contains an arbitrary JSON object and can be used in order to associate data with the geometry. In the case of the developed prototypical implementation, these properties were used to store the (sensor) measurement data. Figure 3 shows an example for a GeoJSON feature, including a polygon, defined by the coordinates of its vertices, and the properties including (sensor) measurement values. The right-hand side of the figure shows a graphical representation of the polygon described by the geometry.

In order to enable efficient storage, processing, and hierarchical aggregation of geospatial data, it is helpful to partition data into buckets. To this end, we make use of a *Discrete Global Grid Systems (DGGS)*. The following definition is provided by the Open Geospatial Consortium (OGC):

**Definition** **3.** *“A DGGS is a spatial reference system that uses a hierarchical tessellation of cells to partition and address the globe. DGGS are characterized by the properties of their cell structure, geo-encoding, quantization strategy and associated mathematical functions” [39].*

A series of discrete global grids represents the spatial reference system of a DGGS. Each of these grids has a finer resolution, as it encompasses an increasing number of cells with respect to its predecessor grid. Since DGGSs cover the whole spherical surface of the earth, they can be used to partition data collected anywhere on the planet.

**Figure 4:**
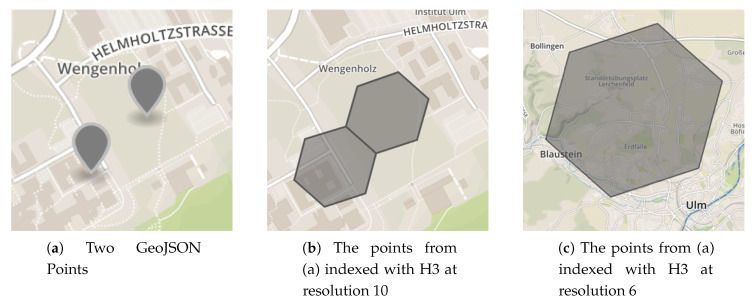
GeoJSON points and their respective H3-indexed polygons at different resolutions (Screenshots from http://geojson.io/).

Furthermore, Uber’s Hexagonal Hierarchical Spatial Index (H3) [40] was used as a DGGS-implementation. Its grid system (a visualization of the grid system can be found on Uber’s website [40]) allows for the representation of the same data efficiently and in differently sized buckets. These characteristics, in turn, are important to aggregate (and visualize) data on different scales. For instance, for the two points depicted in Figure 4a, indexing them with H3 at resolution 10, results in two different indexes with their bounding polygons being next to each other (see Figure 4b), while indexing them with resolution 6, results in one common index including both points (see Figure 4c). Notably, the H3 library allows for a specific geospatial location to determine in which bucket it has to be placed and, inversely, to calculate the boundary of each bucket if its index is known.

## 6. Measurement Context

**Figure 5:**
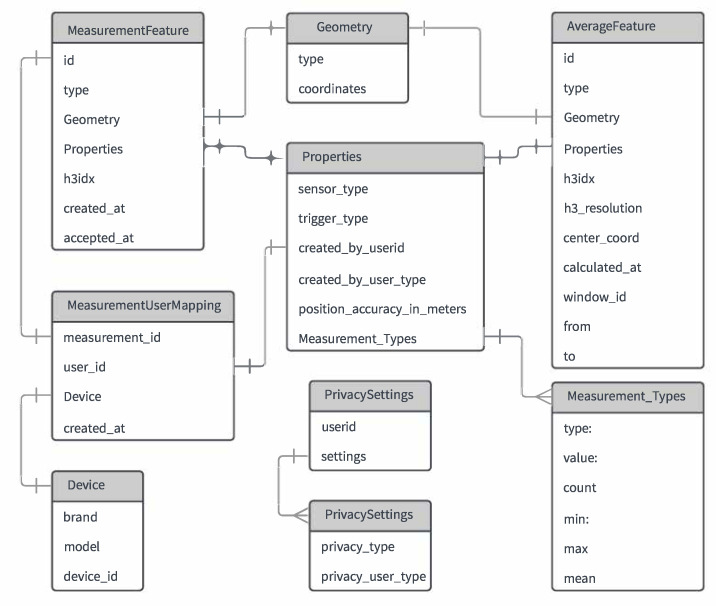
Data model for noise measurements. Reproduced with permission from Kraft et al., In Proceedings of the 2019 IEEE 32nd International Symposium on Computer-Based Medical Systems (CBMS); published by IEEE, 2019 [5].

The measurement context is a decisive aspect and key factor to create a noise level map. This section describes in what way the challenges to efficiently represent and aggregate noise measurements are addressed. At first, the developed document-based (NoSQL) data model for noise measurements that is used for the Measurement Context is shown in Figure 5. GeoJSON Simple Features (see Section 5) are used to model measurements and aggregations in order to provide better compatibility with different geo-libraries and data-storage in MongoDB (https://www.mongodb.com/). Importantly, the latter supports indexing of GeoJSON structures inherently. Technically, the attributes Type, Geometry, and Property are used. A unique id and additionally a geo-index, that is calculated using the H3 library, is assigned to each noise measurement. Properties are used to store the measurement payload, which contains the type of the sensor, the trigger of the measurement, and one or more Measurement_Types (e.g., LAeq, LCPeak, and TWA [41]), amongst other attributes. Each of these types can either contain only the type and a value if they represent a single measurement, or contain minimum, maximum, mean, and count values, if they represent an AverageFeature. The latter is used to store aggregated values for a specific time window in a specific geographical area (i.e., a hexagonal polygon). Any user-related data are stored separately from the measurement data in order to preserve privacy.

Before sending measurements, the user can authenticate himself to the system. The authentication follows a token-based approach based on OAuth 2.0 [42] in order to reduce the transmission of user credentials and enable separation of the authentication in a distributed environment. Users may log in once with their credentials and then receive a signed access token that can be used for all subsequent requests for a certain period of time. Only the Authentication Service (see Section 4) is able to retrieve the user information associated with a token, while other services are only able to validate if the token is valid. Alternatively, users may share their measurements anonymously.

**Figure 6:**
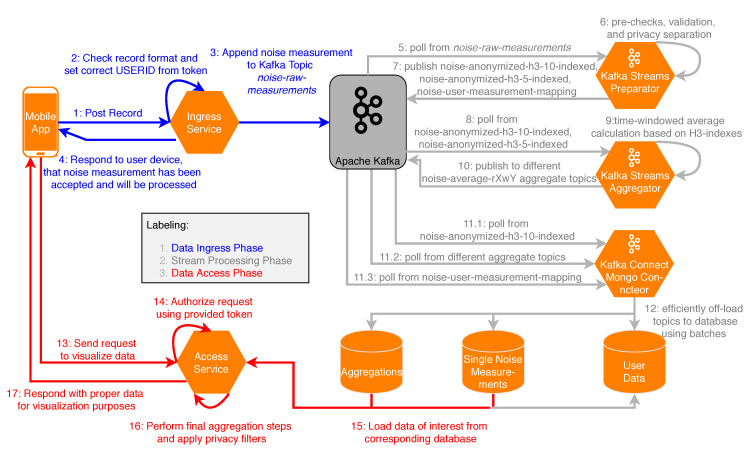
Data flow of noise measurements. Reproduced with permission from Kraft et al., In Proceedings of the 2019 IEEE 32nd International Symposium on Computer-Based Medical Systems (CBMS); published by IEEE, 2019 [5].

**Figure 7:**
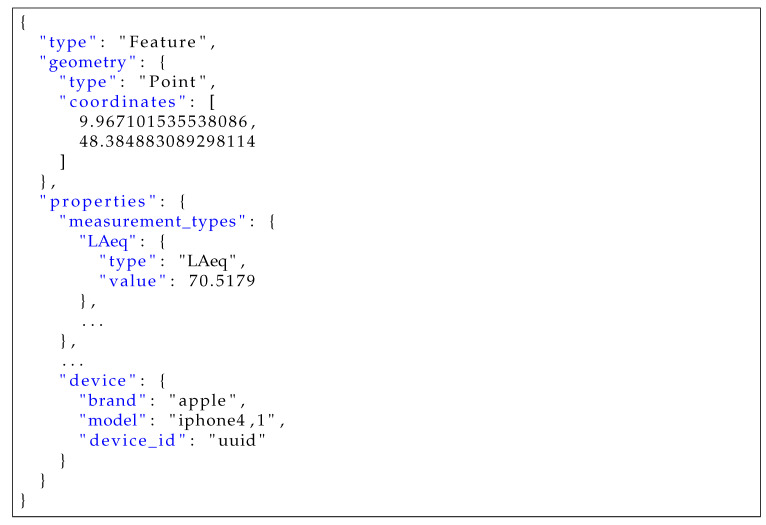
Example GeoJSON object that is sent to the ingress service.

Figure 6 shows the implemented data flow of measurements in the Measurement Context. The Data Ingress Phase consists of Steps 1–4, which are briefly outlined: In Step 1, measurement records from the mobile application are sent to an endpoint of the ingress service, while in Step 2, the ingress service checks records for validity. Additionally, the endpoint handler attaches timestamps as well as a *user_id*, if the user is authenticated. An example for a GeoJSON object, as it is sent to the ingress service, is shown in Figure 7. In Steps 3 and 4, the measurement is published to the Kafka topic noise-raw-measurements, and a confirmation message is sent to the mobile application (if the measurement is valid). Thereafter, the stream processing phase is performed in Apache Kafka in Steps 5–12. In the following, these steps are discussed in more detail.

**Figure 8:**
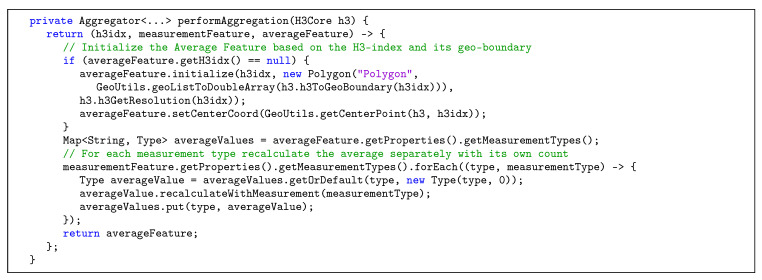
Method that performs the aggregation when a new measurement is added.

**Figure 9:**
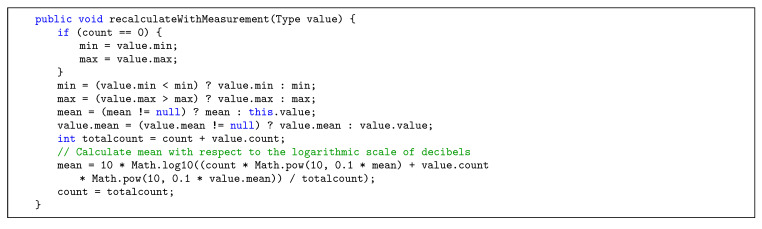
Class method that recalculates the minimum, maximum, and mean values of a Measurement_Type.

The Java library *Kafka-Streams* was utilized in order to publish measurement data to different topics. Table 2 shows the topics currently used in the developed stream processing implementation, together with the key that is used to identify each record and a description for the respective topic. In Steps 5–7, all messages from the noise-raw-measurements topic are processed by the preparator, i.e., measurements are validated and subsequently anonymized for privacy reasons. To be precise, the mapping between users and individual measurements is stored in a separate topic noise-user-measurement-mapping, to which only the user himself has access. This way, all measurements used for the aggregations can be stored without any user data, but the user can still retrieve his own measurements. Furthermore, the coordinates of the measurements are used to calculate H3-indexes with the resolutions 10 (smallest) and 5 (intermediate). The anonymized and indexed data are then published to respective Apache Kafka topics. In Steps 8–10, data from the previous steps are serving as input for the aggregator, in which averages (or other aggregation operations) for the different resolutions and time windows are calculated based on H3-indexes, as shown in Figure 8. A time window is thereby characterized by a window length (e.g., 15 min to allow for reasonably current data) and a retention time. The latter specifies for how long the window is updated retrospectively, if measurements are incoming at a later time (e.g., one day, so that the data from previous days can be considered complete). In the context of noise measurements, minimum and maximum values are determined and averages are calculated with respect to the logarithmic scale of decibels (see Figure 9). Aggregation results are then published to different Apache Kafka topics. In Steps 11–12, the produced results are persisted to a MongoDB with Apache Kafka Connect in order to allow us to efficiently query the data that would otherwise be partitioned across multiple topics in the Kafka Cluster. Finally, in Steps 13–17, data are requested and prepared for visualization in the data access phase, in which the following is processed: In Step 13, a request to the RESTful API of the access service is sent by the mobile application, specifying a H3 resolution, a time window, and a geo-boundary. Optionally, if the request is access-restricted, authorization is performed in Step 14. Furthermore, in Step 15, MongoDB’s geospatial indexes are utilized in order to efficiently load data from the corresponding databases. In Step 16, final aggregations steps are performed if the data is not already present in the database in the requested format (e.g., a time window or resolution that is not pre-aggregated), and privacy filters are applied (e.g., to specify that only the users themselves can see their own raw measurements). Finally, in Step 17, data is delivered to the mobile application, in which it can be used for visualization on a map.

## 7. Proof-of-Concept Prototype

**Figure 10:**
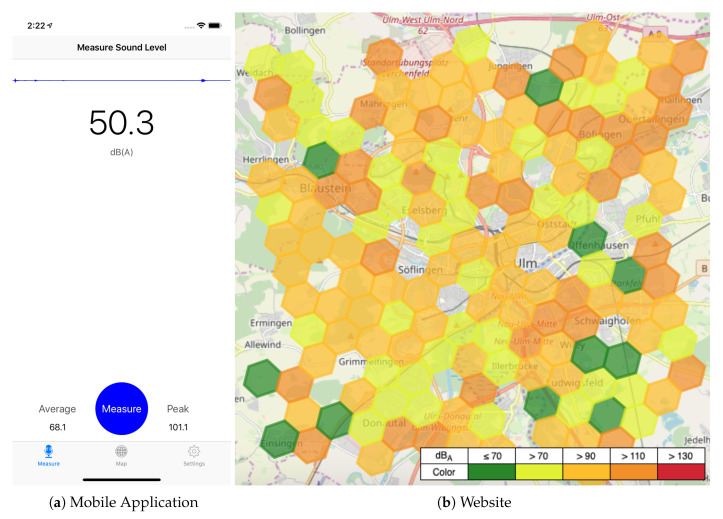
Screenshots of the mobile application and the website showing the noise level map. Reproduced with permission from Kraft et al., In Proceedings of the 2019 IEEE 32nd International Symposium on Computer-Based Medical Systems (CBMS); published by IEEE, 2019 [5].

A proof-of-concept prototype was implemented, which is briefly outlined in the following. Figure 10 shows selected screenshots of the prototype. The current environment sound level in A-weighted decibels (i.e., dB(A)) is continuously displayed to the user of the mobile application (see Figure 10a). Pressing the measure button initiates a noise measurement. For this process, (1) the A-weighted and C-weighted sound levels are tracked over a time period of 30 seconds and cached for further processing, (2) the equivalent continuous A-weighted sound level (LAeq) and the C-weighted peak sound level (LCpeak) [41] are calculated over these cached sound levels, (3) the results are displayed to the user, and (4) finally posted to the backend (either immediately or delayed if the application is currently not able to establish a connection to the server). These measurements are then processed by the backend as described in Section 6. Following this, the mobile application as well as a website (see Figure 10b) can request aggregations for different geo-boundaries, time windows, and zoom levels (i.e., H3 resolutions) through the access service. Utilizing this data, an adequate visualization of the noise exposure in the form of a map can be provided to the user of the mobile application or the website. The noise exposure is thereby indicated by a color gradient between harmful (red) and harmless (green).

At present, users in the region of Ulm, Germany have been acquired to test the proof-of-concept prototype. First feedback indicates that users generally value the application and recognize its benefits. However, regarding the mobile application, so far, solely an iOS mobile application has been implemented. Therefore, an Android application is currently under development. The main reason to only opt for iOS, for the first release, was that sound measurements are more reliable and comparable on iOS regarding the analysis and interpretation of results. Since various hardware vendors with different microphones use Android as operating system, unlike iOS, which only runs on a relatively small number of Apple devices allowing for an easier pre-calibration, the evaluation of retrieved sound levels becomes more complex as it may vary among different Android vendors and would require a mechanism for ad-hoc microphone calibration. However, the completion of an Android application will be a decisive step in order to represent the majority of smartphone users. Furthermore, based on the feedback of the users, the iOS application will be revised and extended by new features. The latter could include incentive mechanisms as well social and communication features, as they were not realized for the first version of the application. In addition, regarding the backend, extensive performance tests are performed in order to evaluate the scalability of the platform, as described in the following section. Finally, an external sensor is currently tested in combination with an Android application that is able to measure even more precise environmental data. In this context, it is also tested how users experience such an external sensor application over time.

## 8. Performance Evaluation

In order to evaluate the performance and scalability of the conceived architecture, *benchmark load tests* were conducted on a running instance of the prototypical implementation of the backend, following guidelines for measuring performance of parallel computing systems [43], and computer systems in general [44]. In the scope of this paper, we evaluate to what extent the performance of the Access Service (i.e., the service used by clients to request measurement data from the backend) under different workloads develops. Other user-centric services like the ingress service are evaluated analogously.

### 8.1. Experimental Setup and Methodology

For the experiments, the prototypical backend was deployed to the *bwCloud*, a cloud provider for scientific and educational purposes by a federation of German universities in the Federal State of Baden-Württemberg in the context of the bwCloud SCOPE project (https://www.bw-cloud.org/en/). The bwCloud provides infrastructure as a service (IaaS) and based on the Open Source software Openstack (https://www.openstack.org/), and the distributed object store and file system Ceph (https://ceph.io/). Terraform (https://www.terraform.io/) is then used as Infrastructure as Code (IAC) tool to configure the cloud resources based on structured text-files. IAC is one of the central parts in building a cloud-native application, as it allows us to maintain, install, and deploy the infrastructure in a reproducible manner [45]. Using this concept, eight virtual machines (i.e., nodes) with each 4 VCPUs and 8 GB RAM running CoreOS 1855.4.0 (http://coreos.com/) were configured for the overall experimental setup. One of these nodes serves as master node for the Open Source software *Rancher* (https://rancher.com/), which is used to facilitate the creation and management of the Kubernetes cluster. Another node, in turn, is used as master node for the Kubernetes cluster itself. The remaining six nodes serve as worker nodes for the cluster. Finally, the developed backend, composed of a total of 14 microservices (see Figure 6, some less relevant services (e.g., for authentication) are omitted for simplicity), was deployed to this cluster setting.

The Open Source testing tool Gatling (https://gatling.io/) for the produced load is used for the actual measurements. Gatling allows us to simulate concurrent users in a resource-saving manner by sending asynchronous messages via non-blocking protocols like HTTP. Previous to the experiment, randomly generated (sound measurement) data for the city of Ulm has been posted to the backend, as displayed in Figure 10b. Benchmark workloads are created by simulating different numbers of concurrent users that (simultaneously) access the stored measurement data for the city of Ulm, and for the last day via the REST API (i.e., the Access Service) utilizing Gatling’s atOnceUsers function. The experiments are run on a single Ubuntu 19.04 machine in the university network that has a stable Internet connection to the bwCloud infrastructure. For each run, the 50% quantiles (i.e., the median) of response times are recorded, as the median is more robust towards outliers as other summary methods like the mean [44]. Note that this median value is considered and handled as a single measurement. The experiments were then repeated and the median of the measurements incrementally recomputed, as well as the confidence intervals (CI) at confidence level 1−α=0.95, as described in [43], determined. A confidence interval provides a measure of accuracy for the experiment, as it bounds the uncertainty of a summarized data set (i.e., the median) of sample data that results from the randomness of non-deterministic measurements. The interval can be interpreted as a 95% probability (i.e., confidence level) that the observed CI contains the true median [44]. Each experiments is repeated *n* times until the CI is within 5% of the median of the respective measurements. Note that n>5 measurements are required to compute the confidence intervals for statistical reasons [44]. To this end, each test run was repeated for n=20 times.

### 8.2. Results

**Figure 11:**
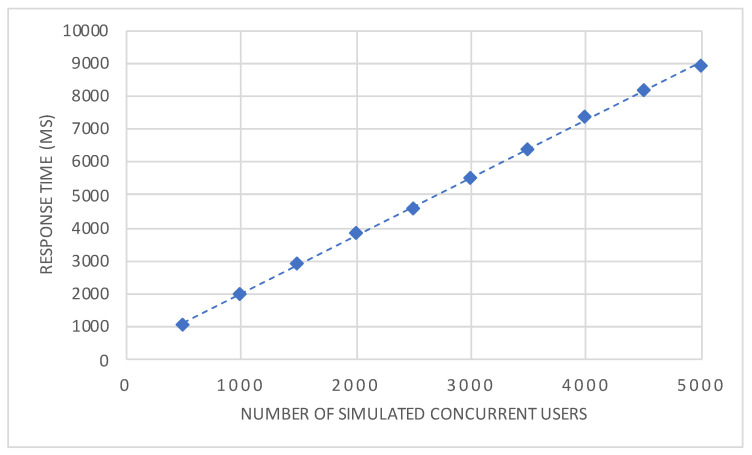
Median of the response time measurements for different numbers of simulated concurrent (simultaneous) users. The dashed line represents an ideal linear trend line. All experiments were repeated 20 times and the confidence intervals (CI) at confidence level 0.95 (1−α=0.959) were within 5% of the respective median.

The results of the experiments are illustrated in Figure 11. The exact medians and confidence intervals of the measurements are shown in Table 3. It can be seen that the response times increase almost linearly, as indicated by the dashed trend line. In order words, these results suggest that the system provides almost ideal linear scaling under different workloads. Regarding the longer response times of up to about 9 seconds, one has to take into account that actual simultaneous requests were used to simulate concurrent users in order to represent extreme situations of workloads. As the proposed architecture is composed entirely out of microservices, horizontal scaling in a large-scale cluster beyond our prototypical experimental setup might lead to a flatter curve of response times. However, this could not be assessed due to a limited amount of (cloud) resources in our experimental setup. Another limitation resulting from the lack of available resources and an suitable load balancer is that the elasticity of the architecture cannot be assessed at the current stage. Furthermore, as the load tests are run from a single machine, no statement can be made for a distributed environment. Generally, the architecture has to prove its suitability in different (mHealth) scenarios. Nonetheless, the experiments, which have been conducted have shown that the proposed architecture is feasible in a running environment and can sustain increasing quantities of load in a scalable manner. Therefore, it can be considered as a first and solid mainstay for the healthcare scenario addressed in this work. Many more considerations and experiments are needed to map the results and architecture to a more generic system. In addition, as medical evidence is always an important aspect in healthcare scenarios, this aspect has to be considered in the light of the presented technical achievements.

Finally, two more aspects must be investigated more in-depth. Beyond the technical performance of the approach, studies will be conducted to capture the demands and experiences of users more properly. Second, based on the aggregation idea of data, it must be evaluated whether other context aspects can be further utilized. For example, there is a difference whether the measurements are accomplished at the beginning or at the end of a day. Or, as another example, the specific city or area, for which the measurements are accomplished, might have different characteristics and needs as other areas. These factors will therefore be considered in future work.

In summary, particularly by taking the promising experimental results into account, the three main technical challenges raised in the introduction are addressed by the proposed technical solution as follows:

- **Scalability and Elasticity**: The architecture is scalable and elastic due to its cloud-native design based on microservices.
- **Efficient Geospatial Processing**: Geospatial data is efficiently processed in the architecture by utilizing stream processing techniques and a DGGS as spatial reference system for data representation.
- **Flexibility**: The architecture is flexible due to the modularity of microservices of the cloud-native design.

## 9. Summary and Outlook

This work presented an approach to create a noise level map using a mobile crowdsensing platform capable of processing noise measurements from a large number of crowd users and their smartphones. Noise exposure for a specific area is thereby indicated by a color coding and different zoom levels. The latter features were made possible through a newly designed measurement context that stores and aggregates noise measurements by developing a sophisticated stream processing pipeline. From a patient point of view, first study results based on a proof-of-concept prototype indicate that users value the platform’s general approach and welcome its ease of use. From the technical point of view, a performance evaluation has been conducted that suggests linear scaling of the conceived architecture under increasing amounts of workloads. However, this work also discussed technical aspects that need to be improved in order to finally provide a feasible approach that can be reliably applied in various practical scenarios. Furthermore, extensive performance tests will be conducted against state-of-the-art architectures to evaluate the scalability of the system in different mHealth and eHealth scenarios again and again. With regard to tinnitus disorder, a noise level map that is based on the discussed approach may be used by patients to avoid burdensome places. Many other useful features for tinnitus patients were revealed when testing the proof-of-concept prototype in practice. For instance, users indicated they could complete a tinnitus-related questionnaire while performing a noise measurement. Using this information, the data of the tinnitus questionnaire can be related to the recorded noise levels to enable users to learn more about the daily fluctuations of their tinnitus. Furthermore, other statements of users indicate that the overall incentive management must be enhanced to motivate users in participating over a longer period of time. From a technical point of view, it was revealed that a complex technical architecture and infrastructure are required for the implementation of the discussed features. The resulting solution, on the other hand, can also be used in other mHealth contexts. For example, the system could be used to measure weather-related factors in the context of migraine. In addition, machine learning (ML) approaches on the data streams have the potential to further improve the system, e.g., by supplementing data sets of areas with sparse measurement contributions. Overall, it was revealed that mobile crowdsensing in the mHealth context is still in its infancy. On the other hand, approaches such as the one presented in this work show that mobile crowdsensing is a promising paradigm for mHealth scenarios. More importantly, the work at hand shows that the combination of medical-driven information science and computer science is an important field that requires more in-depth investigations of interdisciplinary teams.

## Figures and Tables

**Table 1:** Core functions of the platform mapped to bounded contexts. Reproduced with permission from Kraft et al., In Proceedings of the 2019 IEEE 32nd International Symposium on Computer-Based Medical Systems (CBMS); published by IEEE, 2019 [5].

#	Function	Bounded Ctx
1.1	Let users register and authenticate with the backend.	User Identity
1.2	Let users change their password and provide lost-password recovery.	User Identity
1.3	Let users deactivate and delete their user account.	User Identity
2.1	Let users maintain a *User Profile* with personal information.	Social
2.2	Let users join groups and start, follow, and contribute to discussions.	Social
2.3	Provide geospatial relations of groups and discussions.	Social
2.4	Trigger a notification to the user on new contributions in subscribed discussions or subscribed areas of interest.	Social
3.1	Collect measurements provided by smartphones and other IoT-devices and streamline them as a common input stream.	Measurements
3.2	Aggregate the measurements to provide min-, max-, and average values within certain geospatial areas and time-based windows.	Measurements
3.3	Allow geospatial request filtering by specifying the area of interest and time windows.	Measurements
3.4	Allow access to single stored measurements with a pagination like limitation for the number of results.	Measurements
3.5	Provide an API that returns the results in a common geospatial format to allow straightforward visualization features with commonly used frontend technologies.	Measurements
4.1	Track user contributions for authorization of additional functionality and to provide a feature that users can evaluate their progress.	Incentives
4.2	Maintain awards and streaks for certain achievements that motivate users to continue in contributing measurements.	Incentives
5.1	Inform users about certain events via email.	Communication
5.2	Inform users about certain events via push-notifications.	Communication
5.3	Let the user define preferences for the type of events he or she likes to be informed.	Communication
6.1	Manage meta-information about statically deployed sensors.	Sensors

**Table 2:** Kafka topics used in the noise measurement stream processing.

Topic	Key	Description
noise-raw-measurements	created_at	The entrance topic for every measurement.
noise-user-measurement-mapping	userid	Contains mapping objects that relate measurement-id and user-id.
noise-anonymized-h3-10-indexed	H3idx(10)	Contains measurements that are filtered and anonymized. The key is an H3 index of resolution 10, in order to be correctly assigned to partitions used in average aggregation.
noise-anonymized-h3-5-indexed	H3idx(5)	Contains measurements that are filtered and anonymized. The key is an H3 index of resolution 5, in order to be correctly assigned to partitions used in average aggregation.
noise-average-r10w15	H3idx(10)	Contains smaller aggregations in H3-resolution 10 and a window-length of 15 min.
noise-average-r10w60	H3idx(10)	Contains smaller aggregations in H3-resolution 10 and a window-length of 60 min.
noise-average-r5w60	H3idx(5)	Contains larger aggregations in H3-resolution and a window-length of 60 min.
noise-average-r10w1440	H3idx(10)	Contains smaller aggregations in H3-resolution 10 and a window-length of 1 day.
noise-average-r5w1440	H3idx(5)	Contains larger aggregations in H3-resolution 5 and a window-length of 1 day.

**Table 3:** Median of response times and confidence intervals (CI) in milliseconds (ms) for each number of simulated concurrent users.

Number of Concurrent Users	Median of Response Time (ms)	Confidence Intervals (CI)
500	1080.5	[1052 ms, 1115 ms]
1000	2000	[1974 ms, 2027 ms]
1500	2941	[2874 ms, 2972 ms]
2000	3873	[3826 ms, 3937 ms]
2500	4592	[4564 ms, 4662 ms]
3000	5543.5	[5306 ms, 5607 ms]
3500	6414.5	[6264 ms, 6674 ms]
4000	7384	[7174 ms, 7535 ms]
4500	8204	[7904 ms, 8433 ms]
5000	8943	[8795 ms, 9182 ms]
